# Novel Disc Hydrodynamic Polishing Process and Tool for High-Efficiency Polishing of Ultra-Smooth Surfaces

**DOI:** 10.3390/mi9070333

**Published:** 2018-07-02

**Authors:** Bin Lin, Xiang-Min Jiang, Zhong-Chen Cao, Yan Li

**Affiliations:** 1 Key Laboratory of Advanced Ceramics and Machining Technology, Ministry of Education, Tianjin University, Tianjin 300072, China; linbin@tju.edu.cn (B.L.); krancytj@163.com (X.-M.J.); liyan19860615@163.com (Y.L.); 2 Key Laboratory of Mechanism Theory and Equipment Design of Ministry of Education, Tianjin University, Tianjin 300072, China

**Keywords:** disc hydrodynamic polishing, fluid film, computational fluid dynamics, ultra-smooth surface

## Abstract

Nowadays, large aspheric surfaces, including non-rotationally symmetric surfaces, are increasingly used in ground- and space-based astronomical instruments. The fabrication of these surfaces with sub-micrometric form accuracy and nanometric surface finish, especially for hard and difficult-to-machine materials, has always been a challenge to the optics industry. To produce ultra-smooth surfaces efficiently without subsurface damage and surface scratches, a novel disc hydrodynamic polishing (DHDP) process is proposed through the combination of elastic emission machining and fluid jet polishing. Firstly, the polishing tool for DHDP was carefully designed and the feasibility of the proposed method was experimentally verified. The liquid film was found to act as a carrier of abrasive grains between the polishing tool and the polished surface. Next, computational fluid dynamics (CFD) was used to study the effects of process parameters on the slurry film flow in DHDP. Finally, preliminary experiments were conducted to verify the CFD simulations. The experimental data reasonably agree with the simulation results, which show that increasing rotational speed has no influence on the film thickness for the polishing tool without grooves, but leads to increased film thickness for the polishing tool with grooves. Moreover, DHDP can efficiently reduce the surface roughness and acquire ultra-smooth surfaces without subsurface damage and scratches.

## 1. Introduction

Ultra-smooth surfaces are widely used in semiconductors, optics, energy, bio-medicine and aerospace equipment [1]. Specifically, a large aspherical optical surface is the core part of astronomical telescopes and inertial confinement fusion instruments. As the application of ultra-smooth optical surfaces becomes diversified, the demand for improved optical surface quality continues to increase. Moreover, various methods for polishing smooth surfaces are invented and applied in optical glass manufacture. The demand for ultra-smooth surfaces is considerably stricter with the polishing process compared with that for usual optical surfaces [2]. Machining the optical surface without damage to the micro-surface or subsurface structure is often required. A major requirement for the quality surface of optical glass in aerospace and astronomical instruments is thus proposed. The fabrication of these surfaces with sub-micrometre form accuracy and nanometre surface finish, especially for hard and difficult-to-machine materials, has always been a challenge to the optics industry.

Owing to the wide application of ultra-smooth surfaces, modern processing methods are dedicated to research on low damage or non-damage surfaces [3]. Thus, new machining methods have been proposed, such as elastic emission machining (EEM) and fluid jet polishing (FJP) [4], these methods could polish the work surface in micro-structures. Besides, electropolishing (EP) and high-current density electropolishing (HCEP) have also been used for finishing of ultra-smooth metal surfaces [5]. They are commonly applied to the preparation of metal samples with ultra-smooth surfaces such as transmission electron microscopy [6]. However, EP and HCEP are unsuitable for polishing hard and brittle materials and they have a low material removal rate on corrosive resistance materials. The EEM polishing method was presented by Mori et al. in 1987 [7]. This method applies the principle of fluid hydrodynamics to realise the atomic-size machining method on the micro-surface. Su et al. [8,9] studied the hydrodynamic polishing (HDP) process under different lubrication conditions and analysed the machining rate of HDP by using a spherical tool. Results showed that the shear stress of the hydrodynamic zone in the slurry film plays an important role in the removal rate. The shear stress is affected by the spherical tool speed, slurry viscosity and load in different semi-contact and non-contact cases. However, the spherical tool might be damaged because of the limited machining regions with high shear stress. Kim [10] analysed the behaviour of machining fluid, including powder particles, to study the machining mechanism of EEM by using a cylindrical polyurethane wheel. The prediction of material removal rate was acquired by analysing the flow field and the motion of abrasive particles in the machining process. Although the cylindrical wheel could increase the processing area, the polishing efficiency remained limited compared to that using the spherical tool. Hence, satisfying the engineering requirements is still difficult.

In this study, a novel HDP tool is presented, namely, disc hydrodynamic polishing (DHDP) processing [11]. This method combines hydrodynamic theory with polishing theory to achieve the removal of material at atomic-size machining. Furthermore, computational fluid dynamics (CFD) were used to study the effects of process parameters on the slurry film flow in DHDP. Preliminary experiments are conducted to verify the CFD simulation.

## 2. Disc Hydrodynamic Polishing (DHDP)

**Figure 1:**
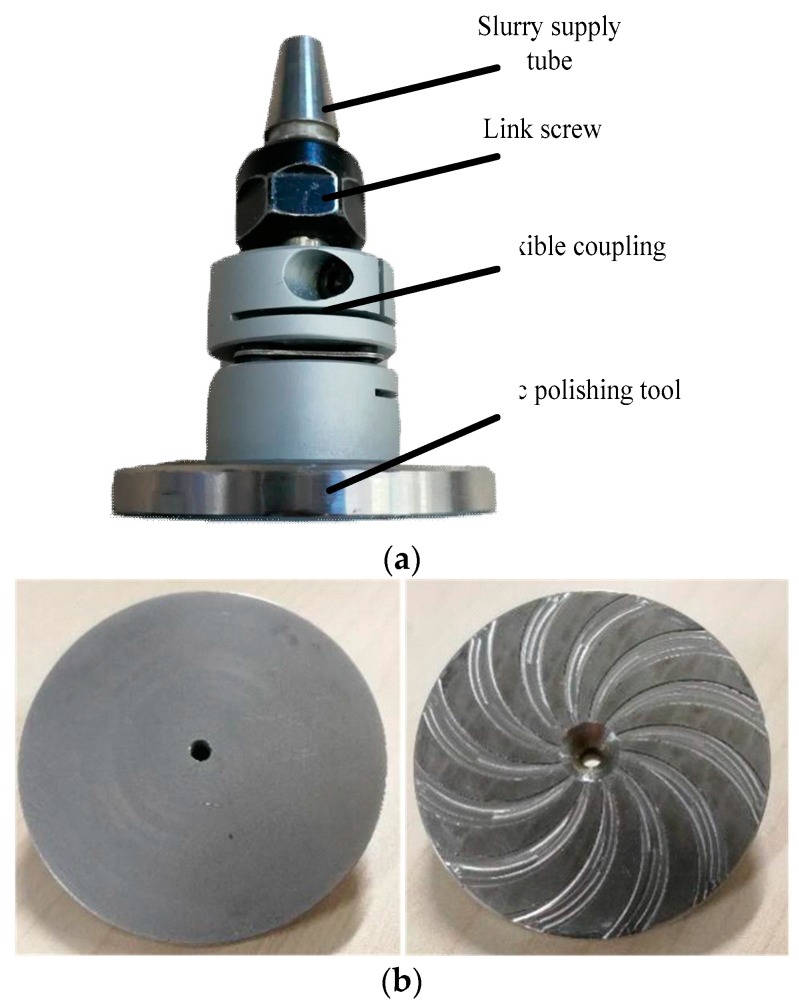
Schematic and physical diagram of a disc hydrodynamic polishing (DHDP) experimental system: (**a**) polishing module, (**b**) disc polishing tool.

The elastic emission machining (EEM) and disc hydrodynamic polishing (DHDP) processing methods are both based on hydrodynamic theory, but the structure of DHDP is relatively different [12,13]. The DHDP polishing tool does not submerge in the slurry, which is supplied through the central pipe at a specific pressure. The DHDP machine consists of a control system, slurry supply system, polishing tool and workpiece. The main component is the polishing module with the disc polishing tool (Figure 1). The tool consists of a motorised spindle, flexible coupling, hose and disc tool. The diameters of the disc tool and centre hole are 50 and 3 mm, respectively. A novel disc polishing tool with a spiral groove was designed based on the hydrodynamic theory of the spiral groove bearing [14]. Twelve spiral grooves were engraved on the disc, and the spiral angle of the groove was 45°. The disc tool was made of metal material and the grooves in the disc were manufactured using a computerized numerical control (CNC) machine.

In the DHDP process, the slurry with abrasive particles was supplied to the central pipe and the outflow from the centre hole of the disc. The polishing disc was designed with different dynamic pressure grooves. These grooves can increase the pressure and velocity of fluid and particles when the tool is driven by the motorised spindle. Then, a fluid film will be formed between the tool and workpiece. The formation of the fluid film is related to the rotational speed of the tool, slurry viscosity, roughness of the workpiece and shape of the disc. Different pressure gradients are formed due to the flow and diffusion of the slurry in the film. Thus, the motion of particles will be accelerated, possibly colliding powder particles with the work surface. The surface material of the workpiece can be removed efficiently. The machining rate on the disc tool will be different due to the pressure gradient in the fluid film. A simulation analysis and preliminary experiment will prove this case.

## 3. Fluid Simulation Analysis Based on Computational Fluid Dynamics (CFD)

In disc hydrodynamic polishing (DHDP), the hydrodynamic analysis in the fluid film is an important measure to investigate the polishing mechanisms in the DHDP process. The slurry is compressed in the gap between the disc polishing tool and the workpiece because of the rotation and axial loading of the tool. Meanwhile, the viscosity of the slurry will increase to resist the compression. As the polishing tool is composed of metal materials, the deformation of the disc tool can be ignored. The fluid film thickness (*h_x_*) can be expressed by the initial fluid film thickness (*h_0_*) and the geometry of the disc tool *g*(*x*,*y*).

(1) $$h\left(x,y\right)=h_{0}+g\left(x,y\right)$$

where *h* is the fluid film thickness. The Reynolds equation is applied to show the relationship among rotational speed, fluid pressure and film thickness [15] as follows:

(2) $$3h^{2}\frac{\partial h}{\partial x}\frac{\partial p}{\partial x}+h^{3}\frac{\partial^{2}p}{\partial x^{2}}+3h^{2}\frac{\partial h}{\partial y}\frac{\partial p}{\partial y}+h^{3}\frac{\partial^{2}p}{\partial y^{2}}=6\mu \omega r\frac{\partial h}{\partial x}$$

**Figure 2:**
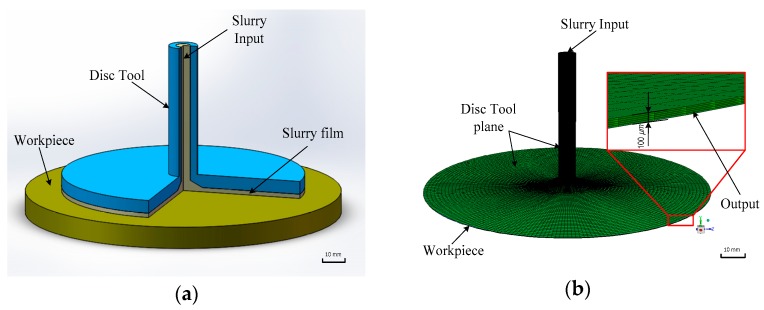
Slurry flow field model of disc hydrodynamic polishing (DHDP). (**a**) The fluid region 3D model. (**b**) The fluid film thickness model with 100 μm.

According to hydrodynamic theory, the fluid between the polishing tool and workpiece is considered to be a Newtonian fluid. The shear stress of the slurry flow on the workpiece can be obtained as

(3) $$\tau =\mu \frac{du}{dh}$$

where *p* is fluid pressure, *μ* is the viscosity of the slurry, *ω* is the angle speed of the disc tool, *r* is the rotational radius, *τ* is the fluid shear stress on the workpiece and *u* stands for the flow velocity calculated from the Reynolds equation. In this section, the CFD model was developed by utilising a central pipe sending slurry with particles and the flow film of the disc polishing tool. In this case, the fluid region 3D model was established, which makes simplifying the complex assembly model possible, as shown in Figure 2a. On the basis of the preliminary study results [9], the thickness of the flow film is probably 50–150 μm. Hence, the thickness of the flow film is set to 100 μm in the CFD model, as shown in Figure 2b.

FLUENT software is used to simulate the flow hydrodynamic characteristics in DHDP. According to hydrodynamic theory, the slurry flow will satisfy the continuity hypothesis when the Reynolds number is less than 2400. The Reynolds number of the polishing slurry flow is approximately 1000–1500, so a laminar model is used to analyse the film layer. The semi-implicit method for the pressure-linked equation is applied to solve the fluid–structure interaction analysis. The polishing tool with different grooves was designed according to the theory of hydrodynamic lubrication. The pressure distribution and velocity of the slurry flow were calculated by using the CFD model.

**Figure 3:**
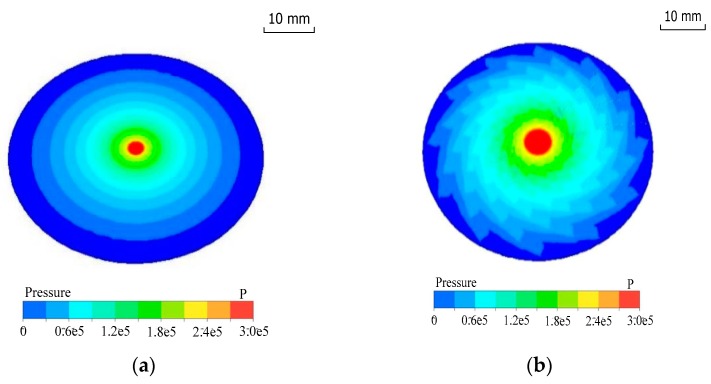
Pressure field of slurry film in the horizontal section. The fluid film thickness of the computational fluid dynamics (CFD) model is 100 μm, the pressure of the slurry inlet is 0.3 MPa and the rotational speed of disc polishing is 3000 rpm. (**a**) Without groove, (**b**) with grooves.

**Figure 4:**
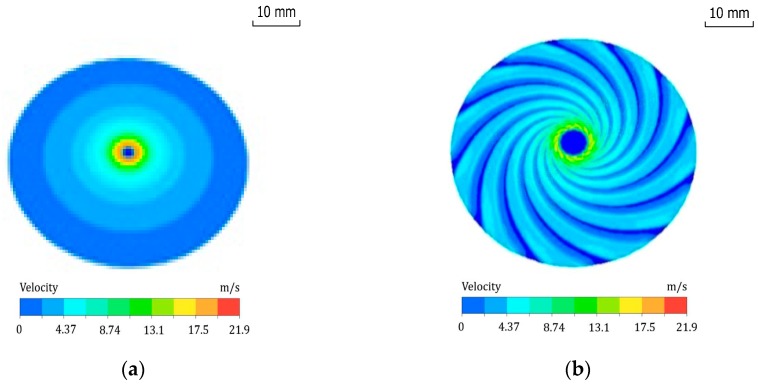
Velocity field of slurry film in the horizontal section. The fluid film thickness of the CFD model is 100 μm, the pressure of the slurry inlet is 0.3 MPa and the rotational speed of disc polishing is 3000 rpm. (**a**) Without groove, (**b**) with grooves.

Figure 3 shows that the fluid pressure is relatively high in the central region, and the pressure will decrease gradually along the radial direction. Notably, the pressure gradient of the disc polishing tool with grooves is smaller than that of the plane polishing tool. Figure 4 illustrates that the velocity gradient distribution is similar to the pressure gradient distribution, except in the stagnant zone in the central region where the flow velocity is relatively low, compared with the surrounding region. As a result, the slurry and the particles will slow down in the centre area. Although the impingement angle in the centre area is large, the low kinetic energy of the particles will cause the surface material to be removed in the ductile mode. After the particles come out of the centre hole, the slurry flow will speed up. The tool with grooves can form a fluid hydrodynamic film in the gap. The area of high pressure and flow velocity will increase. Meanwhile, the grooves can enhance the efficiency of particle collision with the workpiece. The machining rate of material removal is also enhanced.

According to the principle of hydrodynamic pressure polishing, the dynamic pressure and shear stress of the slurry between the tool and workpiece are the main factors affecting polishing in the DHP process [11]. Su [7] proposed that a large shear stress and dynamic pressure will result in a large machining rate. In the DHDP, the slurry fluid was supplied to the central pipe of the disc tool, and the flow film was formed under the high-speed rotation of the tool. The fluid film of the disc polishing tool with screw grooves was analysed by the CFD model. In this simulation, the pressure of the supply slurry was 0.3 MPa and the rotational speed was 3000 rpm. The dynamic pressure and shear stress on the workpiece were investigated under different film thickness (50, 75, 100, 125 and 150 μm). 

**Figure 5:**
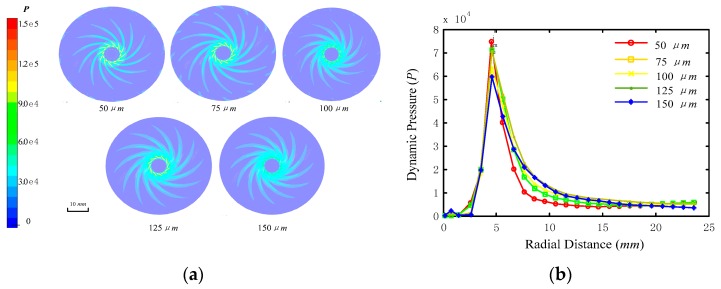
Distribution of dynamic pressure: (**a**) distribution of dynamic pressure under different fluid film thickness, and (**b**) average dynamic pressure along the radial distance of the disc polishing tool.

Figure 5a shows the profiles of the transient dynamic pressure on the flow film under different film thickness. Film thickness has a significant effect on the distribution of the dynamic pressure. The distribution of dynamic pressure is a spiral line shape, and the partial dynamic pressure decreases with the increasing film thickness. However, the area of dynamic pressure is increased with the increase of film thickness. The value of the dynamic pressure was also homogenised along the radial distance on the disc polishing tool, as shown in Figure 5b. The dynamic pressure is minimum at the inlet of the slurry supply. At the distance of 5 mm from the slurry inlet, the dynamic pressure reached a maximum value and then decreased. The smaller the thickness of the fluid film, the greater the dynamic pressure. When the film thickness is small, the peak pressure is high and the width of the dynamic pressure distribution is small. Therefore, the area of the dynamic pressure region is small.

**Figure 6:**
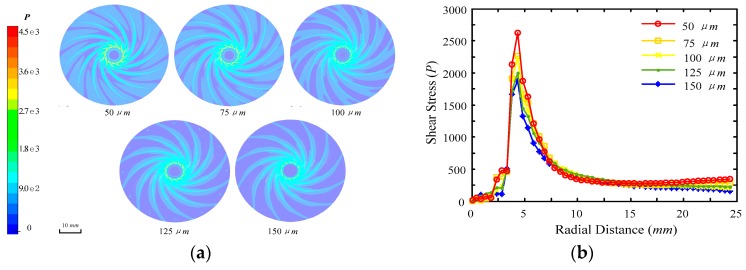
Distribution of shear stress on the workpiece: (**a**) distribution of shear stress under different fluid film thickness and (**b**) average shear stress along the radial distance of the disc polishing tool.

Meanwhile, the shear stress has a similar distribution character as the dynamic pressure shown in Figure 6a. Figure 6b presents the average shear stresses along the radial distance. The shear stress reached a maximum value at the distance of 4 mm from the slurry inlet and decreased rapidly along the radial distance. In comparison with the distribution of dynamic pressure, the value of shear stress and the area region have the same regularities of distribution under different film thickness. The smaller the thickness of the liquid film, the faster the shear stress decreases. The area of the shear stress region is also decreased by the increase of film thickness. The fluid film acts as a carrier of abrasive grains between the polishing tool and workpiece. The results also show that the slurry fluid pressure and velocity are decreased in the radial direction. As the tool with grooves can increase the high-pressure regions, the machining rate can be improved.

## 4. Experimental Analysis in DHDP

**Figure 7:**
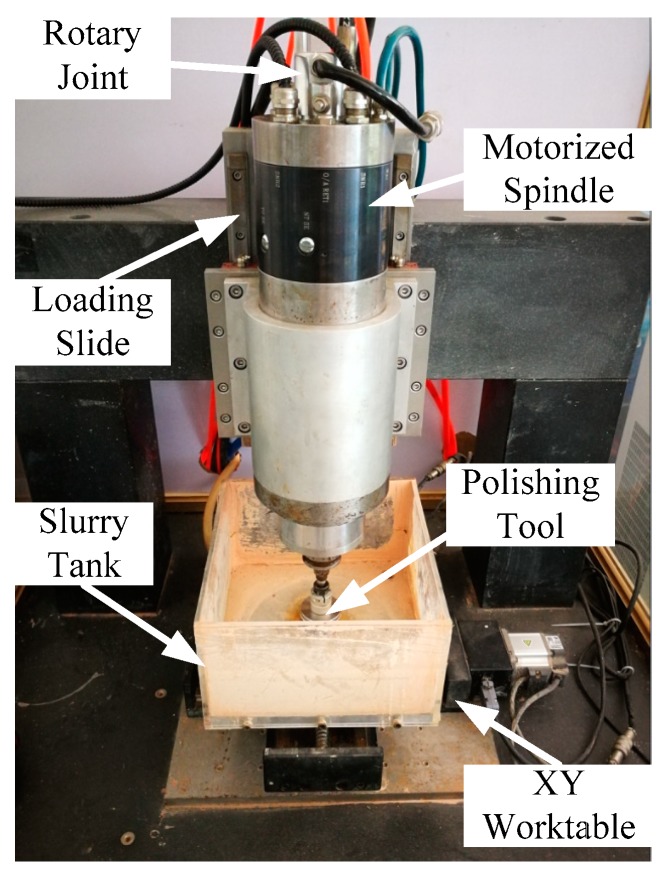
Experimental setup of disc hydrodynamic polishing DHDP.

Figure 7 shows the DHDP experimental setup. The disc tool is fixed on a motorised spindle, and the polishing slurry is injected into the central tube of the spindle. The slurry fluid pressure is provided by a pump and controlled by the computer. A stirred tank is applied to maintain the particle concentration of the slurry during the machining process. The slurry is composed of CeO_2_ particles (400 nm and 1 μm) and water. The disc tool with pressure grooves rotates at the speed range of 1000–5000 rpm under the control of a computerized numerical control (CNC). The tool load is also controlled by an SMC (Tokyo, Japan) air cylinder. 

### 4.1. Experimental Validation of the Computational Fluid Dynamics (CFD) Model

**Figure 8:**
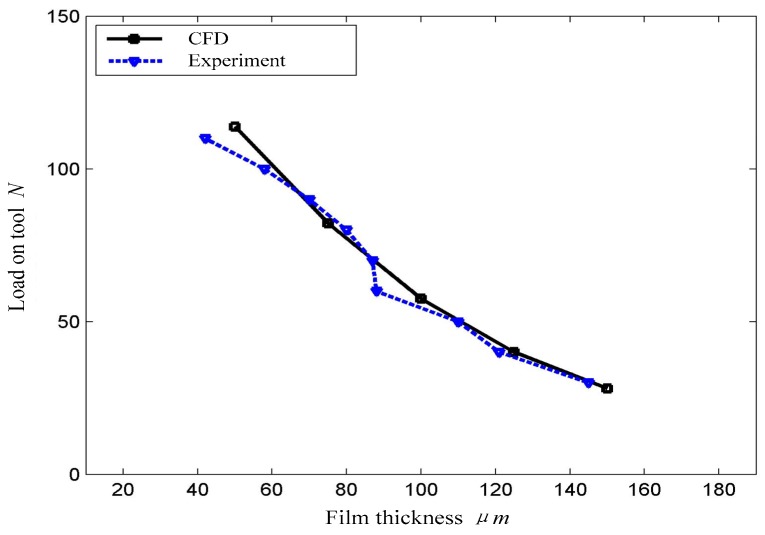
Film thickness experiment and prediction.

According to hydrodynamic theory, the load of the tool can probably affect the slurry thickness. Experiments are conducted to study the relationship of the load and film thickness to prove the existence of the slurry film and its influence on polishing tools. In the experiment, the dial gauge is used to measure the thickness of the slurry film. In combination with the CFD results, the load of the polishing tool can be calculated in different thicknesses of the film, when the speed of the tool is constant. The load control is achieved by adjusting the SMC air cylinder, and the number of dial gauges is recorded in a stable state. Figure 8 shows that the load of the polishing tool decreases as the slurry film thickness increases. The curve of the load–film thickness relationship obtained by CFD simulation and experiment presents a good relationship when the slurry film thickness is in the range of 50–150 μm.

### 4.2. Influence of Rotation on Load–Thickness Curve

The DHDP method that machines the surface via high-velocity particles, which are accelerated by the fluid slurry, influences the surface of the workpiece. Thus, studying how the hydrodynamic film is formed under different rotational speeds of the polishing tool is necessary. In this part of the experiment, the speed of the polishing tool is controlled by the CNC machine. The relation among the thickness values of the film under different rotational speeds of 1000, 1500, 2000, 2500 and 3000 rpm is studied. Moreover, the supply pressure and concentration of the slurry are constant, and the different structures of the polishing tool are considered. The comparative experiment is performed via plane polishing and spiral pressure groove tools.

**Figure 9:**
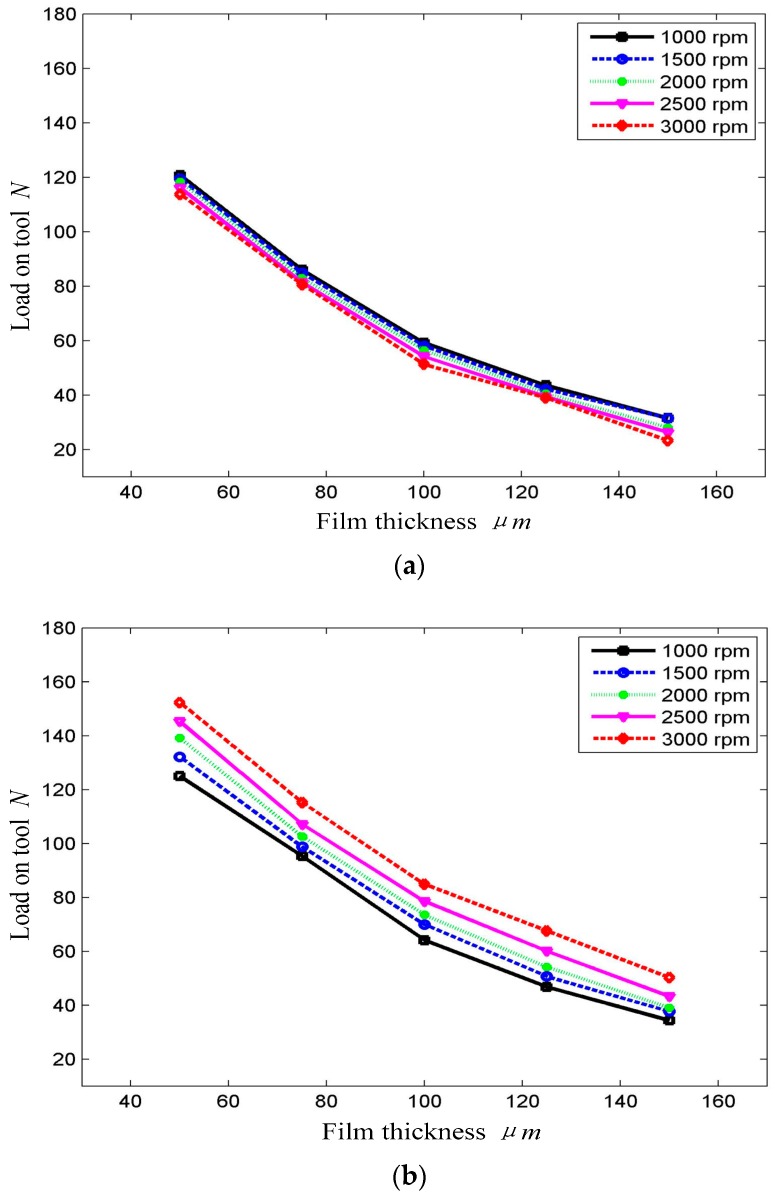
Influence of rotation on load–thickness curve. (**a**) Without grooves; (**b**) With pressure groove.

Figure 9 shows the experimental results. The load on the disc tool without grooves is almost not affected by the rotational speed. On the contrary, the polishing tool with grooves could enhance the hydrodynamic effect of the slurry film. The film thickness will be increased with the increase of tool speed. Thus, the thickness of the slurry film can be controlled by the rotational speed of the tool. Meanwhile, the polishing rate on the surface workpiece will also be controlled.

### 4.3. Surface Finishing by DHDP

**Figure 10:**
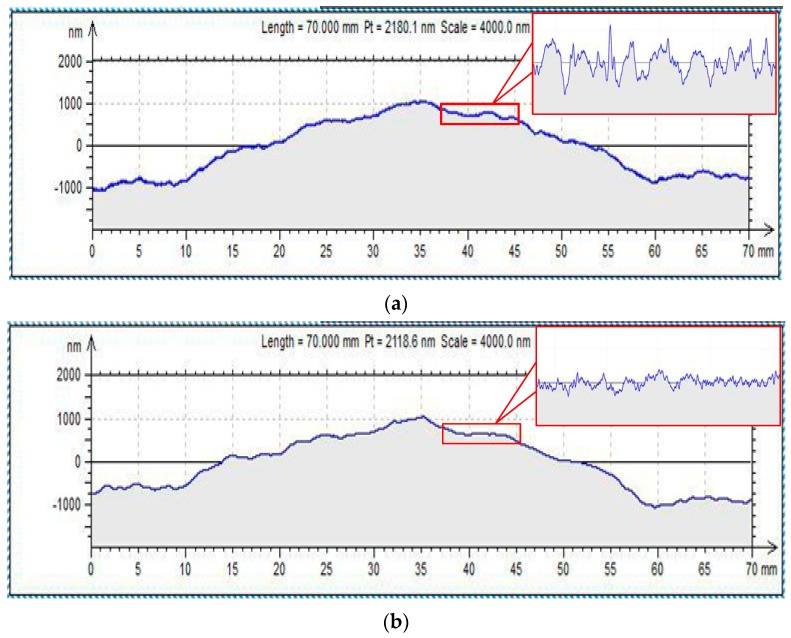
The surface profile of the sample; (**a**) Original and (**b**) polished.

In the practical experiment, the surface of the fused quartz glass is polished. The polishing tool with pressure grooves is applied, the rotational speed is controlled at 3000 rpm and the load in the tool is 80 N. Therefore, the slurry film thickness is approximately 100 μm. After 120 min of processing, the surface roughness of the sample is examined by a light interference instrument (Figure 10). To ensure the accuracy of the measurement results, the two different positions on the sample surface are measured. The respective mean value of Ra and Rz are presented as the characteristics of the surface quality. Figure 10 illustrates that the DHDP process can improve the surface roughness. The maximum height of the profile Rz is reduced from 28.76 nm to 7.81 nm. The surface average roughness (Ra) is reduced from 10 nm to less than 2 nm when the DHDP tool with small particle slurry is used. The material is removed by repeated impact of abrasive particles at the atomic level. Thus, less damage to the subsurface is produced while reducing the surface roughness. The surface quality of the workpiece shows that the DHDP process can efficiently reduce the roughness of the surface and acquire ultra-smooth surfaces without subsurface damage and scratches. In conclusion, the DHDP machining efficiency is higher than EEM in improving the surface roughness.

**Figure 11:**
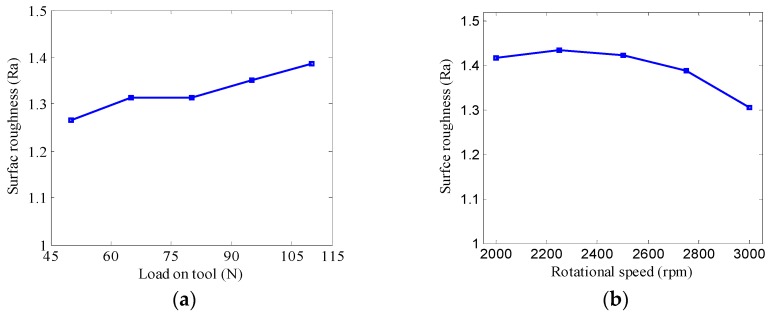
The surface roughness of the polished sample with various process parameters in DHDP; (**a**) Tool load; (**b**) Rotational speed.

To study the effect of the process parameters on the surface roughness of the polished sample for DHDP, a series of experiments are conducted by using different tool load and rotation speeds. The surface roughness is also presented by measuring the different positions, which include the central and peripheral polishing areas. Figure 11a shows that the mean surface roughness of the polished sample is increased with the increasing tool load. According to the above simulation and practical experiments, the film thickness decreases when the tool load is increased. This results in the increasing dynamic pressure and shear stress of the fluid film, and hence produces the increasing material removal rate which in turn deteriorates the surface quality of the polished surface. Figure 11b shows the surface quality is improved with increasing rotation speed. Hence, the results indicate that both the tool load and the rotation speed have significant influence on the surface quality of the polished sample and DHDP is proved to be a promising method that can be successfully used for fabricating ultra-smooth surfaces without subsurface damage and scratches.

## 5. Conclusions

To produce ultra-smooth surfaces efficiently without subsurface damage and surface scratches, a novel disc hydrodynamic polishing (DHDP) is proposed by combining EEM and FJP. Computational fluid dynamics (CFD) are used to study the effects of process parameters on the slurry film flow in DHDP. The CFD results indicate that the distribution of fluid pressure and velocity of the polishing tool with grooves are more uniform and larger than those of the plane disc tool. The thickness of the liquid film also has a significant influence on the distribution of shear stress and the dynamic pressure of the fluid film for the disc polishing tool with spiral screw grooves. The larger the film thickness of the polishing slurry, the larger the distribution area of dynamic pressure but the smaller the mean value of hydrodynamic pressure. The distribution of shear stress has a similar distribution character as the dynamic pressure.

Practical experiments are conducted to verify the CFD simulations. The experimental data reasonably agree with the simulation results. Experimental results show that increasing rotational speed has no influence on the film thickness for the polishing tool without grooves but leads to increased film thickness for the polishing tool with grooves. Moreover, after 120 min of polishing by DHDP, the maximum height of the profile Rz is reduced from 28.76 nm to 7.81 nm and the surface average roughness Ra is reduced from 10 nm to less than 2 nm. The experimental results also indicate that both the tool load and the rotation speed have great influences on the surface quality of the polished sample, and the DHDP process is proved to be a promising method that can be successfully used for fabricating ultra-smooth surfaces without subsurface damage and scratches.
